# Characteristics and diagnoses of acute headache in pregnant women – a retrospective cross-sectional study

**DOI:** 10.1186/s10194-017-0823-1

**Published:** 2017-12-04

**Authors:** Bianca Raffaelli, Eberhard Siebert, Jeannette Körner, Thomas Liman, Uwe Reuter, Lars Neeb

**Affiliations:** 1 Department of Neurology with Experimental Neurology- Charité Universitätsmedizin Berlin, Charitéplatz 1, 10117 Berlin, Germany; 2 Institute of Neuroradiology – Charité Universitätsmedizin Berlin, Charitéplatz 1, 10117 Berlin, Germany; 3 Center of Stroke Research Berlin – Charité Universitätsmedizin Berlin, Charitéplatz 1, 10117 Berlin, Germany

**Keywords:** Headache, Pregnancy, Secondary headache, Red flags, Clinical features

## Abstract

**Background:**

Acute headache is one of the most frequent neurological symptoms in pregnant women. The early diagnosis of underlying secondary conditions has a major influence on patient outcome, especially in emergency settings. However, at the time being no well-established guideline for diagnostic evaluation of acute headache during pregnancy exists. In this study, we aimed to characterize acute headache in pregnant women concerning demographic, clinical, and diagnostic features, and to determine predictors of secondary headache.

**Methods:**

We analysed retrospectively the data of 151 pregnant women receiving neurological consultation due to acute headache at the Charité Berlin between 2010 and 2016. To assess risk factors for secondary headache in these patients we compared multiple anamnestic and clinical features of the primary and secondary headache group.

**Results:**

57.6% of the patients were diagnosed with primary headache, most common migraine and tension type headache. Concerning secondary headaches, the most common aetiologies were infections (29.7%) and hypertensive disorders (22.0%). The primary and secondary headache group were similar in most anamnestic and clinical features. In multivariate logistic regression analysis, secondary headache history [OR 6.6; 95% CI 1.3–33.1], elevated blood pressure [OR 7.2; 95% CI 2.3–22.6], fever [OR 12.1; 95% CI 1.3–111.0] and abnormal neurological examination [OR 9.9; 95% CI 2.7–36.3] represented independent predictors for secondary headache. Regarding additional diagnostic procedures, abnormal thrombocytes, GOT, GPT and CRP, proteinuria, pathologic results of lumbar puncture and neuroimaging were associated with secondary headache.

**Conclusions:**

Secondary headache disorders are common during pregnancy, occurring in over one third of acute headache cases receiving neurological consultation. Most anamnestic and clinical features may not allow a clear distinction between primary and secondary headaches. Clinicians should pay attention to the presence of secondary headache history, elevated blood pressure, fever and abnormal findings in the neurological examination. Additional investigations, including laboratory tests and neuroimaging, are essential for the diagnostic process.

## Background

Primary headache disorders reach a prevalence peak among women of childbearing age due to hormonal influence and particularly oestrogen fluctuations [1, 2]. The prevalence of headache in gravid women has been described to be as high as 35% [3]. At least 5% of pregnancies are affected by de novo headache, meaning either new onset or new type of headache [4]. The most common headache conditions reported during pregnancy are primary headaches such as migraine without aura, followed by tension-type headache and migraine with aura [5]. Primary headache may frequently change its dynamics during pregnancy. Up to three fourth of female patients with tension type headache and migraine with or without aura experience a significant improvement or remission during pregnancy [3, 6]. However, a new onset of primary headache during pregnancy is also possible [7], with a beginning of migraine without aura occurring in 1 to 10% and migraine with aura in up to 14% of the cases [3, 6].

There are many causes of secondary headaches that need to be considered in pregnant women: Pregnancy increases the risk of cerebral venous thrombosis, ischaemic and haemorrhagic stroke, arterial dissection and probably subarachnoid haemorrhage [8, 9]. Furthermore, hypertensive disorders including preeclampsia represent common health issues affecting approximately 5% of the pregnancies worldwide [10]. Pre-existing migraine is associated with a higher possibility of developing preeclampsia [11, 12] and might have an impact on the risk of ischemic stroke during pregnancy [13].

Due to its etiological variety, headache as a symptom challenges physicians in the diagnostic process. Especially in emergency settings, the early diagnosis of underlying secondary conditions has a major influence on patient outcome [9]. However, at the time being no well-established guideline for diagnostic evaluation of acute headache during pregnancy exists [5, 14]. Several “red flags” have been developed for assessing the risk of secondary headache in the normal population, including sudden pain onset, changes in a known headache pattern or focal neurological deficits [5, 15, 16]. Yet there are only few studies addressing these factors in pregnant women of mostly Afro-American or Hispanic ethnicity [17, 18].

To extend the results of the preceding studies, we aimed to characterize in detail acute headache in pregnant women in a German urban population. We focused on a variety of clinical features, diagnostic procedures, as well as final diagnoses. Based on our data, we intend to identify predictors of secondary headaches in pregnant women.

## Methods

### Patients

We analysed retrospectively the medical records of pregnant patients receiving neurological consultation due to acute headache from January 1, 2010 to December 31, 2016 at the Charité hospital in Berlin, Germany. Acute headache was defined as the presence of a new or known headache beginning during the current pregnancy in a previously respectively interictally pain free patient that led to medical consultation*.* We included women older than 18 years with acute headache as a major symptom who presented to the emergency department or received neurological consultation during an in-patient stay. We excluded consultations of women who left the hospital against their physicians’ advice before completing recommended diagnostic procedures. We did not include women in the post-partum period.

### Clinical evaluation

Using the clinical electronic documentation system, we reviewed the data of these patients in detail per chart review.

The collected data included details of the present pregnancy as well as prior pregnancies and potential complications within both. We also gathered information about prior headache diagnoses and other pre-existing neurological, psychiatric, and further medical conditions. We further assessed family (specifically headache) and smoking history. We characterized the current headache based on following features: altered characteristics compared to possible prior headache diagnoses, duration, localization, sudden onset, subjective intensity of the pain (on a verbal rating scale 1–10), pain quality, and dynamics of the pain before the diagnostic procedure.

Associated symptoms included vegetative symptoms, neurological and further autonomic symptoms. Vegetative symptoms were nausea, vomiting, photophobia, and phonophobia. Further autonomic symptoms included conjunctival injection and/or lacrimation, nasal congestion and/or rhinorrhoea, eyelid oedema, miosis and/or ptosis. Neurological symptoms included visual and sensory disturbance, dizziness and/or vertigo, language and motor impairment, and changes of consciousness both quantitative and qualitative. Furthermore, we documented epistaxis and facial paralysis.

We included neurological examination findings in our data compilation and added information given by other departments like gynaecology, internal medicine and otolaryngology. Possible abnormalities documented in the report were further classified based on their causal association to the acute headache. For example, ptosis would be classified as an abnormal finding in neurological examination not related to the acute symptoms when the patient stated that the condition has been pre-existing in this way.

Other physical examination findings included fever and elevated blood pressure. Fever was defined as a body temperature ≥ 38.5 °C or subjective statements of patients about fever. Elevated blood pressure was defined by a single measurement of the systolic blood pressure ≥ 140 mmHg or of the diastolic blood pressure ≥ 90 mmHg.

Diagnostic means were analysed and included laboratory findings, medical imaging and lumbar puncture. A headache expert (LN) confirmed final headache diagnosis after reviewing the collected data based on the classification of the International Headache Society [19].

### Statistical analysis

Statistical analysis was performed using IBM SPSS Statistics, version 24. Subgroup proportions were compared using χ^2^. Using unpaired t test, we stratified results according to the trimester of pregnancy. Logistic regression was used to assessed the correlation between clinical features with a *p* value ≤0.02 in univariate analyses as independent variables and the dependant variable being the final diagnosis secondary headache. Multivariable analyses were restricted to patients without missing values in the respective category; variables were eliminated using a backward elimination procedure. Threshold for statistical significance was defined as a *p* value ≤0,05 for all analyses. The confidence interval was defined as 95%.

## Results

### Demographics, pregnancy details and final headache diagnosis

**Fig. 1:**
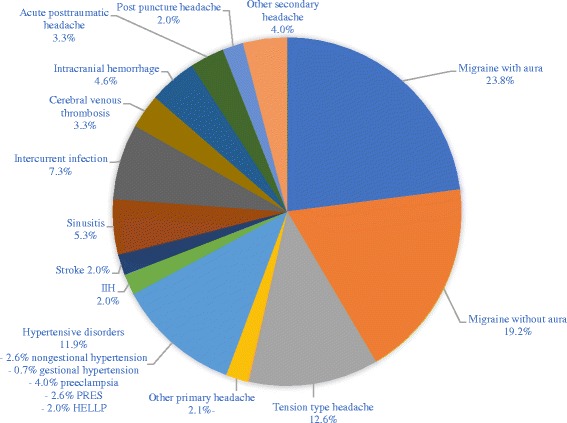
Final headache diagnoses in pregnant women receiving neurological consultation due to acute headache. Percentages may summate to greater than 100% because some patients were given multiple diagnoses. The denominator for all percentages is the size of the total sample (*n* = 151). PRES = posterior reversible encephalopathy syndrome; HELLP = hemolysis, elevated liver enzymes, and low platelet count; IIH = idiopathic intracranial hypertension

Over the 6-year period investigated, we evaluated the clinical features and diagnostic process of acute headache in 151 pregnant women. Diagnoses for acute headache were divided into primary headache (57.6%) and secondary headache (42.4%) (Fig. 1). Within the primary headache group, 41.3% of the women had migraine with aura, 33.3% migraine without aura and 21.8% tension type headache. Secondary headaches were most frequently related to infections, including common viral infections (17.2%) as well as acute sinusitis (12.5%). Other recurrent causes of secondary headache were hypertensive disorders of pregnancy (22.0%), including preeclampsia (9.4%), PRES (6.3%) and HELLP syndrome (4.7%).

In the primary headache group, 50.0% of patients diagnosed with a migraine with aura were in the third trimester (vs. 22.2% in the first and 27.8% in the second trimester). 58.6% of patients diagnosed with a migraine without aura were in the second trimester (vs. 13.8% in the first and 27.6% in the third trimester). However, these differences were not significant. Tension type headache was distributed uniformly during pregnancy with 33.3% in the first, 33.3% in the second and 33.3% in the third trimester.

In the secondary headache group, sinusitis and intercurrent infections were more common in the second trimester (72.7% and 75.0% respectively, not significant), whereas hypertensive disorders occurred more frequently in the last trimester (66.7%, not significant).

The primary and secondary headache groups did not differ by age, number of prior pregnancies, prior deliveries or gestational age. Pregnancy complications occurred significantly more often within the secondary headache group (28.1% vs. 12.6%, *p* = 0.017) (Table 1). The most frequent pregnancy complications were hyperemesis gravidarum (34.5%), followed by recurring hypertensive derailments (17.2%), premature contractions (6.9%), and sickle cell crisis (6.9%).

**Table 1:** Demographics, pregnancy characteristics and medical history of pregnant women receiving neurological consultation due to acute headache

Characteristic	All headache	Primary headache	Secondary headache	*p* value
No.	151	87 (57.6%)	64 (42.4%)	–
Age,years	30.1 (±6.5)	30.4 (±6.3)	29.6 (±6.7)	0.977
Gestations	2.1 (±1.7)	2.3 (±1.9)	1.7 (±1.2)	0.389
Deliveries	0.7 (±1.2)	0.9 (±1.3)	0.5 (±0.9)	0.424
Gestational age, weeks	22.2 (±10.1)	22.5 (±10.3)	21.8 (±9.9)	0.861
Trimester				
First	32 (21.2%)	19 (21.8%)	13 (20.3%)	0.821
Second	63 (41.7%)	35 (40.2%)	28 (43.8%)	0.665
Third	56 (37.1%)	33 (37.9%)	23 (35.9%)	0.802
Complications during current pregnancy	29 (19.2%)	11 (12.6%)	18 (28.1%)	0.017*
Any medical history	51 (33.8%)	27 (31%)	24 (37.5%)	0.406
History of hypertension	5 (3.3%)	2 (2.3%)	3 (4.7%)	0.418
History of venous thromboembolism	3 (2.0%)	2 (2.3%)	1 (1.6%)	0.749
History of autoimmune disease	22 (14.6%)	14 (16.1%)	8 (12.5%)	0.536
History of preeclampsia	4 (2.6%)	2 (2.3%)	2 (3.1%)	0.775
History of gestational diabetes	2 (1.3%)	1 (1.1%)	1 (1.6%)	0.826
Any neurological history	8 (5.3%)	3 (3.4%)	5 (7.8%)	0.237
Any headache history	57 (37.7%)	39 (44.8%)	18 (28.1%)	0.036*
Any primary headache history	51 (33.8%)	39 (44.8%)	12 (18.8%)	0.001*
History of migraine without aura	29 (19.2%)	21 (24.1%)	8 (12.5%)	0.076
History of migraine with aura	14 (9.3%)	12 (13.8%)	2 (3.1%)	0.026*
History of tension type headache	8 (5.3%)	6 (6.9%)	2 (3.1%)	0.307
Any secondary headache history	12 (7.9%)	3 (3.4%)	9 (14.1%)	0.017*

* = statistical significant (*p* < 0.05). Subgroup proportions were compared using χ^2^ test

In the stratified analysis by trimester, pregnancy complications were significantly associated with a secondary headache only in the third trimester (*p* < 0.001).

### Medical history

33.8% of the women reported any medical condition, excluding neurological and psychiatric conditions. Autoimmune diseases, most frequently Hashimoto’s thyroiditis, were the most common medical comorbidity (14.6%), followed by respiratory diseases (3.3%) and non gestional hypertension (3.3%). Rates of all reported conditions were similarly present in primary and secondary headache groups.

History of any psychiatric condition was reported in 5.3% of the patients, referring in all cases to depression, in one case combined with an eating disorder (0.7%), in another case with an anxiety disorder (0.7%). Psychiatric comorbidities were more common in patients with primary headache (8.0%) vs. secondary headache (1.6%) but the difference was not significant (*p* = 0.079).

Prior neurological conditions, excluding headache disorders, were reported in 5.3% of the patients. The most frequent condition was polyneuropathy (1.3%). There was no significant difference between the primary and the secondary headache group (*p* = 0.237) (Table 1).

In the stratified analysis by pregnancy trimester, no significant differences emerged.

### Headache history

Any history of headache, both primary and secondary, was present in 37.7% of the patients. 33.8% of the women suffered of a primary headache disorder, most common migraine without aura (19.2%), migraine with aura (9.3%) and tension type headache (5.3%). 7.9% reported a prior secondary headache, most frequently caused by sinusitis (4.0%). Any history of headache was more common in patients with primary headache (44.8% vs. 28.1%, *p* = 0.036). Women with primary headache reported significantly more often a history of primary headache (44.8% vs. 18.8%, *p* = 0.001) and especially of migraine with aura (13.8% vs. 3.1%, *p* = 0.026). Women with secondary headache reported significantly more frequently a history of secondary headache (14.1% vs. 3.4%, *p* = 0.017).

In the stratified analysis by pregnancy trimester, a prior primary headache correlated significantly with a current primary headache during first trimester (*p* = 0.033), while a prior secondary headache was significantly associated with a current secondary headache during third trimester (p = 0.033).

In cases with a positive headache history, 86% of the patients stated that the current attack was different from the known headache pattern. Differences were reported in associated symptoms (38.6%), increased attack severity (37.5%), localisation (26.8%), duration (23.2%) and frequency (8.9%).

48.8% of patients with a known migraine without aura were diagnosed with the same diagnosis (*p* < 0.001), while 20.7% developed an aura. 71.4% of patients with a known migraine with aura, received the same diagnosis in the current attack (p < 0.001), while 14.2% reported no aura in the index headache. However, most commonly patients with a diagnosed migraine with aura experience attacks with and without aura. De novo migraine without aura was diagnosed in 15 patients (9.9%). A new onset of a migraine with aura was diagnosed in 26 patients (17.2%).

Further details about headache history are given in Table 1.

### Acute headache attack features

A detailed overview of the headache characteristics investigated here is shown in Table 2. In short, patients with primary headache were more likely to report a side predominance of the pain (39.1% vs. 18.8%, *p* = 0.007). A dynamic pain progression was more common in patients with secondary headache (37.2% vs. 19.3%, *p* = 0.046). Visual and sensory disturbance were significantly more often reported within the primary headache group (40.2% vs. 20.3%, *p* = 0.009; 31.0% vs. 10.9%, *p* = 0.003).

**Table 2:** Acute headache attack features in pregnant women receiving neurological consultation due to acute headache

Feature	Missing	All headache	Primary headache	Secondary headache	*p* value
Pain duration >24 h	21 (13.9%)	74 (56.9%)	37 (50.7%)	37 (64.9%)	0.104
Sudden onset	–	8 (5.3%)	2 (2.3%)	6 (9.4%)	0.055
Side predominance	–	46 (30.5%)	34 (39.1%)	12 (18.8%)	0.007*
Throbbing character	58 (38.4%)	36 (38.7%)	24 (44.4%)	12 (30.8%)	0.182
Subjective pain ≥8/10	56 (37.1%)	33 (34.7%)	16 (28.1%)	17 (44.7%)	0.095
Progressive dynamic	51 (33.8%)	27 (27.0%)	11 (19.3%)	16 (37.2%)	0.046*
Any vegetative symptoms	–	93 (61.6%)	55 (63.2%)	38 (59.4%)	0.631
Nausea/Vomiting	–	81 (53.6%)	48 (55.2%)	33 (51.6%)	0.660
Phonophobia	–	25 (16.6%)	18 (20.7%)	7 (10.9%)	0.111
Photophobia	–	32 (21.2%)	22 (25.3%)	10 (15.6%)	0.151
Syncope	–	9 (6.0%)	7 (8.0%)	2 (3.1%)	0.207
Visual disturbance^a^	–	48 (31.8%)	35 (40.2%)	13 (20.3%)	0.009*
Sensory disturbance^b^	–	34 (22.5%)	27 (31.0%)	7 (10.9%)	0.003*
Language impairment	–	17 (11.3%)	12 (13.8%)	5 (7.8%)	0.251
Vertigo or dizziness	–	16 (10.6%)	7 (8.0%)	9 (14.1%)	0.235
Motoric impairment	–	10 (6.6%)	6 (6.9%)	4 (6.3%)	0.875
Change of consciousness	–	5 (3.3%)	1 (1.1%)	4 (6.3%)	0.083
Seizures	–	3 (2.0%)	0 (0.0%)	3 (4.7%)	0.041*
Autonomic symptoms	–	6 (4.0%)	3 (3.4%)	3 (4.7%)	0.700

* = statistical significant (*p* < 0.05). Subgroup proportions were compared using χ^2^ test

^a^ = 58.3% scintillating scotoma, 31.3% blurred vision

^b^ = 73.8% unilateral hypoesthesia, 17.6% unilateral paresthesia

The most common visual disturbance was a scintillating scotoma (58.3%), followed by blurred vision (31.3%). Sensory disturbance referred mostly to unilateral slowly spreading numbness (73.5%) or paresthesia (17.6%).

Seizures were present in 4.7% of the patients with secondary headache compared to none in the primary headache group (*p* = 0.041).

Considering only patients presenting during the first trimester, subjective pain ≥8/10 and progressive pain dynamics were significantly associated with a secondary headache diagnosis (p = 0.041 and *p* = 0.037, respectively). In the second trimester, side predominance and sensory disturbance correlated significantly with a primary headache (*p* = 0.025 and *p* = 0.032, respectively). In the third trimester, progressive pain dynamics occurred more often in secondary headache (*p* = 0.014), while nausea and visual disturbance were more frequent in primary headache (*p* = 0.035 in both cases).

### Clinical examination

The physical examination was abnormal in 9.6% of the cases, significantly more often within the secondary headache group (15.9% vs. 4.8%, p = 0.025). Patients with secondary headache had significantly more frequently elevated blood pressure (31.7% vs. 8.4%, *p* < 0.001) and fever (14.1% vs. 1.1%, *p* = 0.002). Pathological neurological examination findings were detected significantly more often within the secondary headache group (35.9% vs. 11.5%, p < 0.001) (Table 3).

**Table 3:** Clinical examination findings in pregnant women receiving neurological consultation due to acute headache

Feature	Missing	All headache	Primary headache	Secondary headache	*p* value
Abnormal medical examination	5 (3.3%)	14 (9.6%)	4 (4.8%)	10 (15.9%)	0.025*
Elevated blood pressure	5 (3.3%)	27 (18.5%)	7 (8.4%)	20 (31.7%)	0.000*
Fever	–	10 (6.6%)	1 (1.1%)	9 (14.1%)	0.002*
Abnormal neurological examination	–	33 (21.9%)	10 (11.5%)	23 (35.9%)	0.000*
Abnormal neurological examination referable to acute symptoms	–	26 (17.2%)	6 (6.9%)	20 (31.3%)	0.000*

* = statistical significant (*p* < 0.05). Subgroup proportions were compared using χ^2^ test

In the stratified analysis by pregnancy trimester, no significant differences emerged during the first trimester. During the second trimester, fever and pathological results in the neurological examination correlated significantly with a secondary headache (*p* = 0.020 and *p* = 0.018, respectively). During the third trimester, an abnormal physical examination (*p* = 0.006), elevated blood pressure (*p* = 0.001), and abnormal results in the neurological examination (*p* = 0.003) were significantly associated with a secondary headache.

### Clinical variables independently associated with secondary headache

Using binomial logistic regression, we analyzed the independent associations of demographic and clinical variables that differed significantly between both headache groups. Secondary headaches were associated with known prior secondary headache, elevated blood pressure, fever and neurologic examination abnormalities. Primary headaches were more likely in patients with reported visual disturbance (Table 4).

**Table 4:** Multivariate logistic regression analysis of clinical and demographics variables associated with secondary headache in pregnant women

Variable	OR (95% CI)	*p* value
Complications during current pregnancy	2.2 (0.7–6.8)	0.155
Prior primary headache	0.5 (0.2–1.3)	0.147
Prior secondary headache	6.6 (1.3–33.1)	0.021*
Side predominance	0.5 (0.2–1.3)	0.141
Visual disturbance^a^	0.3 (0.1–1.0)	0.048*
Sensory disturbance^b^	0.4 (0.1–1.4)	0.154
Elevated blood pressure	7.2 (2.3–22.6)	0.001*
Fever	12.1 (1.3–111.0)	0.028*
Abnormal neurological examination referable to acute symptoms	9.9 (2.7–36.3)	0.001*

* = statistical significant (*p* < 0.05)

^a^ = 58.3% scintillating scotoma, 31.3% blurred vision

^b^ = 73.8% unilateral hypoesthesia, 17.6% unilateral paresthesia

### Additional diagnostic procedures

Any additional diagnostic procedure was performed in 96.0% of the cases: Blood tests were conducted in 94.7%, urine analysis in 57.0%, neuroimaging in 50.3% and lumbar puncture in 13.2% of the cases with no differences between the primary and secondary headache group.

Any blood value outside the reference range was found in 88.8% of the cases. Abnormal thrombocytes (16.4% vs. 5.0%, *p* = 0.025), abnormal GOT (35.3% vs. 2.3%, *p* < 0.001), abnormal GPT (17.5% vs. 2.0%, *p* = 0.006) and abnormal CRP (58.9% vs. 30.4%, *p* = 0.001) were found significantly more often in the secondary headache group.

Proteinuria was detected in 16.3% of the patients, significantly more frequently in patients with secondary headache (25.6% vs. 8.5%, *p* = 0.032). All other findings did not differ significantly between both headache groups (Table 5).

**Table 5:** Additional diagnostic performed in pregnant women presenting with acute headache

Feature	Missing	All headache	Primary headache	Secondary headache	*p* value
Any additive diagnostic	–	145 (96.0%)	82 (94.3%)	63 (98.4%)	0.193
Additive blood test	–	143 (94.7%)	81 (93.1%)	62 (96.9%)	0.307
Abnormal blood test	8 (5.3%)	127 (88.8%)	69 (85.2%)	58 (93.5%)	0.116
Abnormal Hb	10 (6.6%)	72 (51.1%)	43 (53.8%)	29 (47.5%)	0.465
Abnormal Erythrocytes	10 (6.6%)	46 (32.6%)	27 (33.8%)	19 (31.1%)	0.774
Abnormal Leucocytes	10 (6.6%)	64 (45.4%)	33 (41.3%)	31 (50.8%)	0.258
Abnormal Thrombocytes	10 (6.6%)	14 (9.9%)	4 (5.0%)	10 (16.4%)	0.025*
Abnormal Quick	35 (23.2%)	2 (1.7%)	2 (3.1%)	0 (0.0%)	0.198
Abnormal PTT	35 (23.2%)	3 (2.6%)	1 (1.5%)	2 (3.9%)	0.422
Abnormal D-Dimers	103 (68.2%)	33 (68.8%)	17 (60.7%)	16 (80.0%)	0.155
Abnormal Creatinin	25 (16.6%)	13 (10.3%)	7 (9.9%)	6 (10.9%)	0.848
Abnormal Na	44 (29.1%)	12 (11.2%)	5 (8.5%)	7 (14.6%)	0.319
Abnormal K	44 (29.1%)	9 (8.4%)	4 (6.8%)	5 (10.4%)	0.500
Abnormal GOT	73 (48.3%)	13 (16.7%)	1 (2.3%)	12 (35.3%)	0.000*
Abnormal GPT	60 (39.7%)	7 (7.7%)	1 (2.0%)	7 (17.5%)	0.006*
Abnormal CRP	26 (17.2%)	54 (43.2%)	21 (30.4%)	33 (58.9%)	0.001*
Proteinuria	65 (43.0%)	14 (16.3%)	4 (8.5%)	10 (25.6%)	0.032*
Additive lumbar punction	–	20 (13.2%)	9 (10.3%)	11 (17.2%)	0.220
Pathologic LP results	131 (86.8%)	4 (20.0%)	0 (0.0%)	4 (36.4%)	0.043*
Neuroimaging	–	76 (50.3%)	40 (46.0%)	36 (56.3%)	0.212
Pathologic neuroimaging results	75 (49.7%)	29 (38.2%)	5 (12.5%)	24 (66.7%)	0.000*

* = statistical significant (p < 0.05). Subgroup proportions were compared using χ^2^ test

Pathological results of lumbar puncture were detected in 20.0% of the performed analyses and only in patients with secondary headache (36.4% vs. 0.0%, *p* = 0.043).

Of the 76 patients who underwent neuroimaging, 38.2% had pathologic results, significantly more frequently in the secondary headache group (66.7% vs. 12.5%, *p* < 0.001). All patients undergoing neuroimaging received a magnetic resonance imaging (MRI), 2 (2.6%) both a computed tomography scan and a MRI. Most frequent pathological findings were intracranial bleeding (28.6%), cerebral venous thrombosis (23.8%), and PRES (19.0%).

In the stratified analysis by pregnancy trimester, an abnormal CRP correlated significantly with a secondary headache in the first and second trimester (*p* = 0.017 and *p* = 0.009, respectively). In the second trimester, also abnormal thrombocytes had a significant association with a secondary headache (*p* = 0.019). During the third trimester, abnormal GOT (*p* < 0.001), abnormal GPT (*p* = 0.019), and proteinuria (*p* = 0.049) correlated significantly with a secondary headache.

In every trimester, there was a strong correlation between pathologic neuroimaging results and secondary headache diagnosis (p < 0.001 in first and second trimester, *p* = 0.004 in third trimester).

## Discussion

We reviewed the neurological consultations of 151 pregnant women who presented with acute headache, most of them during second and third trimester. The majority of our sample was diagnosed with primary headache disorders, most frequently migraine with aura, without aura and tension type headache. However, 42% of the women were found to have a secondary headache, most commonly headache attributed to infections and hypertensive disorders of pregnancy. Infectious diseases occurred more frequently during the second trimester of pregnancy, while hypertensive disorders were more common during the third.

Pregnant women with secondary headache presented more often with pregnancy complications, positive secondary headache history, progressive pain dynamic, seizures, abnormal medical examination, elevated blood pressure, fever and abnormal neurological examination. In blood lab tests, abnormal thrombocytes, elevated transaminases and CRP were associated with a secondary headache diagnosis. Furthermore, proteinuria, pathologic findings in the cerebrospinal fluid and pathologic neuroimaging results correlated with a secondary headache. In multivariate logistic regression analysis, secondary headache history, elevated blood pressure, fever and an abnormal neurological examination resulted as independent risk factors for secondary headache.

Pregnant women with primary headache reported more frequently a history of primary headache, a side predominance of pain as well as visual and sensory disturbance, likely driven by migraine and migraine aura [20]. In fact, the most commonly described visual and sensory deficits were typical aura symptoms, such as scintillating scotoma and slowly spreading unilateral hypoesthesia.

 Only few previous studies focused on the clinical evaluation of headache in pregnant women. Robbins et al. characterized demographic and clinical features of pregnant women presenting with acute headache in a predominantly Hispanic and Afro-American population [17]. Ramchandren et al. evaluated medical imaging results in pregnant women with emergent headache. Multiparous Afro-American women constituted the majority of their cohort [18]. However, to the best of our knowledge, this is the first study to assess headache features in pregnancy in a primarily Caucasian population.

Besides the differences in ethnicity, the study group assessed by Robbins et al. had more prior pregnancies and live births. The number of final secondary headache diagnosis was slightly lower than in our study. The most common primary headache disorders coincided with our reported results, namely migraine and tension type headache [17]. Within the secondary headache group, the authors found a higher number of hypertensive disorders of pregnancies. This is probably due to the fact that Afro-American ethnicity is a known risk factor for the development of hypertensive disorders [17, 19]. In the study by Ribbins et al., final diagnosis of primary headache correlated with history of headache [17]. Our results extend this finding by showing that only a previous primary headache correlated with a primary headache diagnosis, whereas a known secondary headache could be considered as a risk factor for a current secondary headache. A detailed differentiation of previous headache history could lead to a better differential diagnostic assessment.

MR imaging is the preferred imaging method for evaluating headache in pregnant women, as it involves no exposure to ionizing radiation [1]. In the study of Robbins et al., neuroimaging was performed in almost 90% of the cases, with a 18% rate of pathological imaging findings [17]. In the analysis of Ramchandren et al., only pregnant women receiving neuroimaging were considered and an underlying headache etiology was revealed in 27% of pregnant women suffering from acute headache [18]. In our study, only 50% of the patients had neuroimaging and 38% of them had pathologic results. The higher rate of pathologic results despite lower neuroimaging incidence could indicate a more detailed clinical preselection of cases. Considering the limited assess to 24 h MRI-imaging in some areas and the economic costs, further research should examine which anamnestic and clinical aspects are crucial in determining the decision to order neuroimaging procedures in pregnant women.

Our study is the first one to demonstrate a possible association between laboratory findings and secondary headache during pregnancy. Low thrombocytes and elevated transaminases, especially during the third trimester, were significantly associated with secondary headache. Such laboratory abnormalities are common in pregnancy-associated hypertension und increase with disease severity [21]. Multiple abnormal values, as occurring in HELLP syndrome, are also associated with maternal and perinatal morbidity and mortality [21]. Furthermore, abnormal CRP values as marker for infections were also more common within the secondary headache group.

One of the main strengths of our study is the large sample size, the broad variety of analyzed clinical details and confirmation of headache diagnosis by a headache specialist. However, our study has some limitations. We characterized cases of acute headaches in a mainly Caucasian sample, yet we cannot provide specific information about the ethnic background of our patients since this information is not routinely acquired in our institutional records. Still, we can assume that our study provides adequate information about an urban population in Europe. About 70% of Berlin’s population are ethnic German, other ethnic influences come mainly from Southern Europe and the Middle East [22]. Further limitations include the retrospective character of the study and, due to that, some missing details of headache features. Moreover, the study was underpowered to detect differences between groups in rarely occurring features, e.g. pregnancy complications. As we analyzed only headaches, we did not consider isolated auras, which may also represent an issue in pregnant women. In fact, migraine aura is the most frequent condition leading to a focal neurological deficit during pregnancy and visual deficits during pregnancy are almost in two third of the cases related to a migraine aura [20]. Due the cross-sectional nature of the study, we were not able to follow up the headache development during the remaining pregnancy. We cannot exclude that some headache diagnosed as a primary form over the course revealed as a secondary headache, especially in those women without additional diagnostic procedures. Furthermore, we were not able to provide information about delivery and child outcome, as most women did not give birth at our hospital. The index headache was the first headache attack during pregnancy that led to a neurological consultation in our hospital. We cannot completely exclude previous consultations at other hospitals and cannot provide additional information about previous attacks in the same pregnancy.

## Conclusions

Headache is a common complaint in pregnant woman. Distinguishing benign headache from ominous secondary changes is of great importance, and can be challenging especially in an emergency setting. We could show that secondary headaches are common during pregnancy, occurring in over one third of pregnant women presenting to the hospital with acute headache. Our findings show that clinical features of secondary and primary headache do not necessarily differ and are in many cases not sufficient to rule out a possible threat to the mother or unborn child.

Diagnostic vigilance should be highlighted in presence of previous history of secondary headache, progressive pain, seizures, fever, high blood pressure and pathological findings in neurological examination. These symptoms can be considered as predictors for secondary headache in pregnant women. However, attack features alone cannot adequately discriminate between primary and secondary headache. Additional diagnostic tests leading to final diagnosis include blood, urine and cerebrospinal fluid examination as well as neuroimaging. In presence of the above mentioned “red flags”, low thresholds for additional diagnostic procedures are justified.

## Abbreviations

CI: Confidence interval
CRP: C-reactive protein
GOT: Glutamic oxaloacetic transaminase
GPT: Glutamate-pyruvate transaminase
Hb: Hemoglobin
HELLP: Hemolysis, elevated liver enzymes, and low platelet count
IIH: Idiopathic intracranial hypertension
K: Potassium
LP: Lumbar puncture
MRI: Magnetic resonance imaging
Na: Sodium
OR: Odds ratio
PRES: Posterior reversible encephalopathy syndrome
PTT: Partial thromboplastin time
